# The biology of seed discrimination and its role in shaping the foraging ecology of carabids: A review

**DOI:** 10.1002/ece3.7898

**Published:** 2021-09-15

**Authors:** Khaldoun A. Ali, Christian J. Willenborg

**Affiliations:** ^1^ Plant Sciences Department College of Agriculture and Bioresources University of Saskatchewan Saskatoon SK Canada

**Keywords:** Carabid (ground) beetles, foraging, postdispersal seed predation, seed bank, weed biocontrol, weed seeds

## Abstract

Species of carabid (ground) beetles are among the most important postdispersal weed seed predators in temperate arable lands. Field studies have shown that carabid beetles can remove upwards of 65%–90% of specific weed seeds shed in arable fields each year. Such data do not explain how and why carabid predators go after weed seeds, however. It remains to be proven that weed seed predation by carabids is a genuine ecological interaction driven by certain ecological factors or functional traits that determine interaction strength and power predation dynamics, bringing about therefore a natural regulation of weed populations. Along these lines, this review ties together the lines of evidence around weed seed predation by carabid predators. Chemoperception rather than vision seems to be the primary sensory mechanism guiding seed detection and seed selection decisions in carabid weed seed predators. Selection of weed seeds by carabid seed predators appears directed rather than random. Yet, the nature of the chemical cues mediating detection of different seed species and identification of the suitable seed type among them remains unknown. Selection of certain types of weed seeds cannot be predicted based on seed chemistry per se in all cases, however. Rather, seed selection decisions are ruled by sophisticated behavioral mechanisms comprising the assessment of both chemical and physical characteristics of the seed. The ultimate selection of certain weed seed types is determined by how the chemical and physical properties of the seed match with the functional traits of the predator in terms of seed handling ability. Seed density, in addition to chemical and physical seed traits, is also an important factor that is likely to shape seed selection decisions in carabid weed seed predators. Carabid responses to seed density are rather complex as they are influenced not only by seed numbers but also by trait‐based suitability ranks of the different seed types available in the environment.

## Graphical abstract

The study elucidates the possible mechanisms that bring weed seeds and carabid predators together, and then, it highlights the key ecological factors that determine strength and dynamics of seed predation interactions.

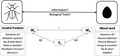

## 1 INTRODUCTION

Predation is one of the fates many weed seeds succumb to either on the mother plant or after seed dispersal (Crawley, 2000). Predispersal seed predation, generally speaking, is largely carried out by specialist species usually belonging to insect orders of Diptera, Lepidoptera, Hymenoptera, and Coleoptera (Kolb et al., 2007). The magnitude of predispersal seed predation pressure and how effective it might be in bio‐regulating weed populations remain difficult to generalize. Some studies, for instance, have shown that predispersal weed seed predation could significantly depress seedling recruitment of some shrubs and perennials (Anderson, 1988; Moles et al., 2003; Moles et al., 2003). Likewise, studies on annual weeds revealed that larvae of the specialized micro‐lepidopterans *Coleophora lineapuvella* (Chambers) and *Scrobipalpa atriplicella* (Fischer von Röslerstamm) could destroy large numbers of the seed heads of *Amaranthus retroflexus* L. and *Chenopodium album* L., respectively, in corn and soybean fields in Eastern Canada (Nurse et al., 2003). However, the seed‐destructive effects of those predispersal predators were sporadic and remained rather low throughout the season and showed high variability across locations and years (DeSousa & Swanton, 2003; Nurse et al., 2003). These findings led the authors to suggest that predispersal weed seed predation alone is unlikely to bring about significant suppression of annual weeds in arable fields. By contrast, the presence of the gall midge *Clinodiplosis cilicrus* (Kieffer) larvae in the flower heads of *Centaurea cyanus* L. in a field study in France was found to be associated with a four‐fold reduction in seed numbers, and about 40% drop in seed viability. This suggests *C. cilicrus* larvae can potentially depress populations of *C. cyanus* in the field (Koprdova et al., 2015). However, the presence of gall midge larvae was not associated with visible seed damage but rather reduced ovule fertilization. The effect therefore cannot be attributed to genuine seed predation but to fertilization disruption by consuming resources necessary for successful fertilization or repelling pollinators (Koprdova et al., 2015).

Postdispersal weed seed predation, on the other hand, is carried out by a wide range of seed predators that span both vertebrate and invertebrate taxa (Moles et al., 2003a; Moles et al., 2003b; White et al., 2007). By and large, arable fields harbor rich faunae of invertebrates that exhibit postdispersal weed seed‐feeding habits (Lundgren, Ellsbury, & Prischmann, 2009). Species of ground (carabid) beetles (Carabidae: Coleoptera), crickets (Gryllidae: Orthoptera), ants (Formicidae: Hymenoptera), and slugs (Gastropoda: Mollusca) are usually among the main postdispersal weed seed predators in agricultural fields of temperate regions (Lundgren & Harwood, 2012; van der Laat et al., 2015). Slugs remain the least studied group, but evidence from field and laboratory studies suggests that contributions of slugs to postdispersal weed seed predation are likely minor (Cardina et al., 1996; Dudenhoffer et al., 2016). Ants, by contrast, engage in a wide range of ecological interactions with seeds of weed species, spanning from mutualism to antagonism (Gammans et al., 2005). Harvester ants, for instance, were found to remove large numbers of weed seeds from fields of dryland cereals in some regions of Europe (Torra et al., 2016; Westerman et al., 2012). The impactful weed seed removal activities of harvester ants remain limited to warm and dry regions of the temperate climates, however, as other competing granivores such as carabid beetles are not highly active in such regions (Evans & Gleeson, 2016). This leaves carabid beetles and crickets as the two dominant invertebrate postdispersal seed predatory groups in warm and wet regions of temperate climates (Carmona et al., 1999; Lundgren et al., 2013). The role of crickets as postdispersal weed seed predators of annual weeds is well documented, but they are difficult to trap and not widely studied (Lundgren, Ellsbury, & Prischmann, 2009; White et al., 2007). By contrast, carabid species are widely distributed, easy to trap, and show high species richness in arable fields (Gaines & Gratton, 2010; Irmler, 2003). Therefore, carabid weed seed predators will be the main species of focus in this review.

## 2 WEED SEED CONSUMPTION BY CARABID PREDATORS

Carabid beetles, generally speaking, function as epigaeic polyphagous predators in agroecosystems (Lovei & Sunderland, 1996). Adults of carabid species can show diurnal or nocturnal activities depending on their habitat and are voracious predators able to consume close to their body weight of food each day (Kromp, 1999; Tuf et al., 2012). Predatory carabids prey upon a wide array of agricultural pests including aphids, dipteran eggs and midges, lepidopteran caterpillars, springtails, earthworms, and slugs (Clark et al., 1994; Floate et al., 1990; Kromp, 1999; Suenaga & Hamamura, 1998). In addition, numerous species of carabid predators are known to feed on seeds of weed species after seed shed (Carbonne et al., 2020; Kulkarni et al., 2015a; Lundgren, Ellsbury, & Prischmann, 2009). Some species of *Harpalus* sp. and *Amara* sp. can even attack weed seeds on the mother plant prior to seed shed (Sasakawa, 2010a). Data from field studies have shown that carabid beetles in some cases are responsible for removing between 65%–90% of certain weed seeds from arable fields each year (see Table 1 for a summary of selected studies documenting postdispersal removal rates of weed seeds by carabid beetles and other invertebrate groups). Overall, the literature promotes carabid beetles as effective natural agents capable of destroying large numbers of weed seeds in arable fields (Bohan et al., 2011). Such elevated seed mortality pressures imposed by carabid weed seed predators are likely to bring about considerable disruption in abundance, distribution, and demography of weed communities in arable fields (Jeanzen, 1971; Jermy, 1984; Davis et al., 2011).

**TABLE 1:** A selection of studies summarizing field data of seed removal rates of some weed species from arable fields by postdispersal weed seed predators

Weed species	Average seed removal rates	Study duration	Seed predatory group	Crop	References
*Ambrosia artemisifolia* L., *Amaranthus retroflexs* L., *Casia obtusifolia* L., *Datura stramonium* L.	4.2%–4.8% day^−1^	5 weeks	Carabid beetles, crickets, and ants	Corn and soybean	Brust and House (1988)
*Alopecurus myosuroides* Huds., *Bromus sterilis* L., *Avena fatua* L.	1.43%–7.2% day^−1^	1 month	Invertebrates	Grassy margins of cereal fields	Povey et al. (1993)
*Chenopodium album* L., *Echinochloa crusgalli* (L.) Beauv.	22%–28% day^−1^	2 years	Invertebrates	Corn, soybean, and wheat	Cromar et al. (1999)
*Digitaris sanguindis* (L.) Scop.	11% day^−1^	2 weeks	Invertebrates	Corn	Menalled et al. (2000)
*Setaria faberi* Herm.	58% 2 days^−1^	3 months	Carabid beetles and crickets	Wheat with red clover	Davis and Liebman (2003)
*Ambrosia trifida* L.	57%–70% year^−1^	12 months	Carabid beetles	Corn (no‐till)	Harrison et al. (2003)
*Stellaria media* L., *C. album*, *A. fatua*	38%–74% year^−1^	2 years	Carabid beetles and mice	Organic cereal fields	Westerman et al. (2003)
*Abutilon theophrasti* Medik.	17% 2 days^−1^	2 years	Carabid beetles, crickets, and prairie deer mice	Corn and soybean	Westerman et al. (2005)
*A. theophrasti*	32% 2 days^−1^	2 years	Carabid beetles, crickets, and prairie deer mice	Corn, soybean, triticale, and alfalfa‐alfalfa	Westerman et al. (2005)
*Polygonum aviculare* L., *Sinapis arvensis* L., *S. media*, *C. album*	35% week^−1^	2 weeks	Carabid beetles	Spring barley	Mauchline et al. (2005)
*Setaria faberi* Herm., *A. theophrasti*	16%–30% day^−1^	4 months	Invertebrates	Cereals and legumes	Heggenstaller et al. (2006)
*Lolium rigidum* Gaudin	57% 2 weeks^−1^	3 months	Ants and invertebrates	Postharvest cropping field	Jacob et al. (2006)
*A. fatua*	42% 2 weeks^−1^	3 months	Ants and invertebrates	Postharvest cropping field	Jacob et al. (2006)
*Raphanus raphanistrum* L.	45% 2 weeks^−1^	3 months	Ants and invertebrates	Postharvest cropping field	Jacob et al. (2006)
*Panicum dichotomiflorum* Mchx., *C. album*	10%–90% day^−1^	3 months	Carabid beetles	Corn (organic field)	Menalled et al. (2007)
*Taraxacum officinale* Weber	34%–40% year^−1^	2 years	Carabid beetles and isopods	Grassland	Honek et al. (2009)
*S. faberi*, *A. trifida*, *A. theophrasti*	11% day^−1^	4 weeks	Carabid beetles	Potato	Gaines and Gratton (2010)
*Amaranthus retroflexus* L.	5% day^−1^	4 months	Carabid beetles	Potato	Gaines and Gratton (2010)
*Lolium multiflorum* Lam.	11.5%–29.8% 18 days^−1^	18 days	Carabid beetles	Winter wheat	Baraibar et al. (2011a)
*Avena ludoviciana* Durieu	33%–63% 6 weeks^−1^	6 weeks	Invertebrates	Barley	Noroozi et al., 2012
*Hordeum spontaneum* (K. Koch) Thell.	27%–33% 6 weeks^−1^	6 weeks	Invertebrates	Barley	Noroozi et al. (2012)
*Galium spurium* L.	60%–70% 2 days^−1^	2 years	Harvester ants *Messor barbarus* L.	Dryland cereals	Westerman et al. (2012)
*Lolium rigidum* Gaud	90%–100% 2 days^−1^	2 years	Harvester ants *M. barbarus*	Dryland cereals	Westerman et al. (2012)
*Papaver rhoeas* L.	90% 2 days^−1^	2 years	Harvester ants *M. barbarus*	Dryland cereals	Westerman et al. (2012)
*Bromus diandrus* Roth	20% 2 days^−1^	2 years	Harvester ants *M. barbarus*	Dryland cereals	Westerman et al. (2012)
*Viola arvensis* Mur., *Capsella bursa‐pastoris* (L.) Medik., *A*. *myosuroides*	30% week^−1^	5 weeks	Carabid beetles	Winter cereals	Trichard et al. (2013)
*C. album*	53%–65% 2 days^−1^	3 months	Carabid beetles and crickets	Corn and soybean	Van der Laat et al. (2015)
*Amaranthus tuberculatus* (Moq.)	80%–85% 2 days^−1^	3 months	Carabid beetles and crickets	Corn and soybean	Van der Laat et al. (2015)
*Brassica napus* L.	42.3%–69.7% week^−1^	3 weeks	Carabid beetles	Canola	Kulkarni et al. (2016)
*S. arvensis*	41%–58.9% week^−1^	3 weeks	Carabid beetles	Canola	Kulkarni et al. (2016)
*Thlaspi arvense* L.	16.2%–28.3% week^−1^	3 weeks	Carabid beetles	Canola	Kulkarni et al. (2016)
*Avena sativa* L. (as a substitute for weed seeds)	78%–100% day^−1^	3 weeks	Harvester ants *M. barbarus*	Dryland cereals	Torra et al. (2016)

Larval carabids, on the other hand, are also predaceous but their feeding ecology remains poorly studied. Based on the evidence to date, carabid larvae seem to feature seed‐feeding habits similar to adults (Sasakawa, 2010b; Sasakawa et al., 2010). For instance, larvae of *Amara* sp. and *Harpalus* sp. were reported to consume large numbers of weed seeds in laboratory feeding bioassays (Saska, 2015; Saska & Jarosik, 2001). Weed seed consumption by larval carabids could reach fairly high levels in some cases, similar in number to levels reported for adults (Klimes & Saska, 2010). Such findings are intriguing but should be approached with caution as they were observed in laboratory feeding experiments only. As yet, no direct measurements of weed seed removal rates by larval carabids have been carried out in the field.

**FIGURE 1:**
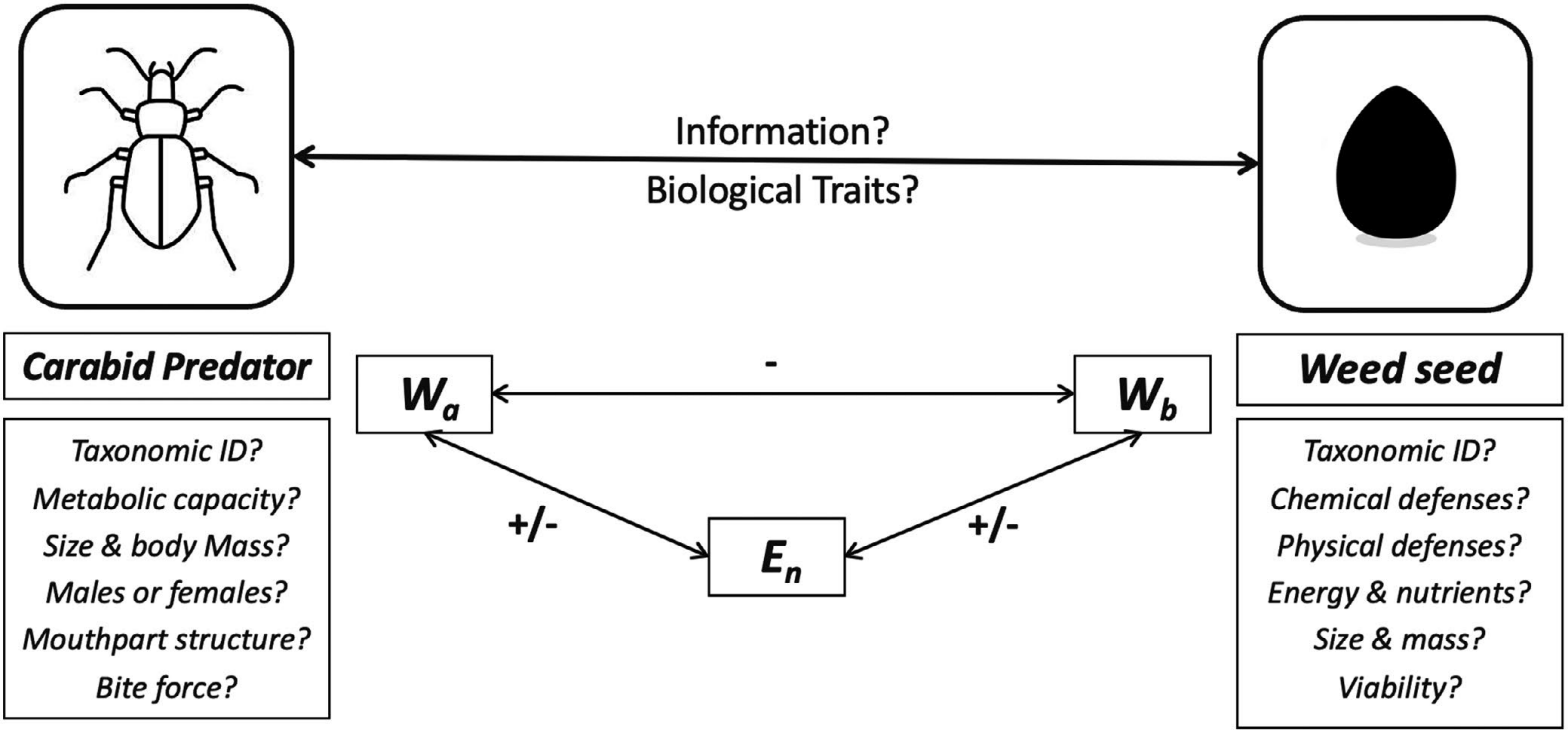
A hypothetical ecological matrix illustrating the possible ecological factors that are likely to influence weed seed predation dynamics in agroecosystems—question marks indicate a possible but not widely understood role for the proposed factor. *W_a_*, fitness of the carabid predator; *W_b_*, fitness of the weed species; *E*, various environmental factors that possibly influence predation dynamics

It remains enigmatic why carabid beetles choose to consume weed seeds or how seed‐feeding preferences evolve given the abundance and diversity of prey carabids have access to in arable fields (Booij et al., 1994; Lovei & Sunderland, 1996). These two big questions have surrounded seed predation ecology for a long time, spurring a lot of speculation without reaching definitive conclusions (see Figure 1). Traditionally, seed‐eating carabid beetles were considered opportunistic feeders that fell into two main groups; omnivores and granivores (Fawki & Toft, 2005; Talarico et al., 2016). This dichotomous grouping suggests that seeds are not primary to diets of omnivorous carabids and would be mostly consumed upon random encounter rather than carabids foraging for seeds specifically (Cardina et al., 1996; Lovei & Sunderland, 1996). Studies on seed‐feeding habits have revealed that the distinction between omnivory and granivory in carabid species is ambiguous, however, as the two feeding habits often overlap (Fawki et al., 2003; Fawki & Toft, 2005). For example, some carabid species originally proposed to feed strictly on animal prey were found to also include considerable amounts of weed seeds in their diets (Carbonne et al., 2020). Similarly, multiple species of carabids within the genera *Amara* sp. and *Harpalus* sp. previously were assumed to subsist on diets composed mainly of seeds. However, many of these species turned out to include nontrivial amounts of animal prey in their diets as well (Frank et al., 2016; Loughride & Luff, 1983). Strict granivory on the whole is quite rare among carabids, and carabid species that feed on seeds exclusively can be found in only a few genera like *Ophonus* sp., *Ditomus* sp., *Dixus* sp., and *Carterus* sp. (Talarico et al., 2016). Together, these findings indicate that the vast majority of seed‐eating carabid species actually belong in the mixed‐feeding omnivorous group, whereas true granivory remains scarce. Molecular gut content analyses spanning both mixed‐feeders and carabid species more specialized toward seed feeding in Europe found high levels of seed DNA from weedy species in the guts of both groups (Frei et al., 2019). The unexpected and significant presence of weed seed DNA in guts of mixed‐feeding carabids contradicts the opportunistic seed predation reasoning introduced in the early carabid literature. Instead, seed foraging behaviors in carabid seed predators are more likely driven by specific biological needs that are likely to influence predation dynamics in certain ways (Carbonne et al., 2020; Davis et al., 2011; Headrick & Goeden, 2001).

The type of biological needs that are likely to underlie seed‐feeding habits and the choice of suitable seeds in carabids remain unknown. Multiple attempts to uncover the key ecological factors that render seed of certain weed species more likely to incur elevated carabid attacks in arable fields have not been very fruitful thus far (Kulkarni et al., 2015a). Large‐scale weed seed predation studies show that predation pressures fluctuate considerably in space and time and do not follow general trends or patterns (Hatton et al., 2015; Honek et al., 2003; Hulme, 1998; Jacob et al., 2006; Kulkarni et al., 2017a). Although some correlations between carabid activity density and regulation of soil seed banks could be established in a few large‐scale studies (Bohan et al., 2011; Carbonne et al., 2020), such relationships were absent in several small‐scale studies (Petit et al., 2014; Saska et al., 2008). Such mixed results, in addition to the correlational nature of the evidence, preclude establishing a general and definitive functional link between activity density of seed predatory carabids and the regulatory pressures they impose on soil seed banks. Mechanistic studies would be more informative in this regard, with a better ability to elucidate how and why weed seeds are attacked by carabid predators. Studies carried out within the mechanistic paradigm would help to tease out the key factors that underlie correlational relationships in the seed predation literature. They would also help to bridge the knowledge gaps that still remain in weed seed predation ecology (van Regenmortel, 2004; Dean & Thornton, 2007; Baskett, 2012). Therefore, in addition to studying seed predation ecology on large scales in the field, seed predation should also be studied on small scales and at deep mechanistic levels. This way, numerous confounding factors could be filtered out, and pseudo‐replication issues could be avoided to uncover the functional aspects of weed seed predation interactions and identify the ecological factors that determine interaction strength and power predation dynamics (Davies & Gray, 2015; Hurlbert, 1984). Uncovering the mechanistic aspects of seed predation ecology is likely to improve the design and conduct of weed seed predation studies on larger scales (Denny & Benedetti‐Cecchi, 2012). Along these lines, we attempt to review the weed seed predation literature in the following sections to synthesize the current knowledge around postdispersal weed seed predation, but in a mechanistic context. Our aim is to tie together the mechanistic results from isolated studies with ecological processes that will provide an improved synthesis of how the biology and ecology of these mechanisms shape weed seed consumption. The discussion will be mainly reserved for adult carabids, and information about larval carabids will be provided, where relevant.

## 3 THE ECOLOGICAL PROCESSES OF POSTDISPERSAL WEED SEED PREDATION BY CARABID BEETLES

### 3.1 Seed and carabid predator co‐occurrence in space and time

Seed predation requires synchronicity between seed availability and predators’ activities both in space and time. Seeds of annual weeds are usually shed into arable fields at certain times, but can persist in the seed bank for extended periods (Baskin & Baskin, 1998). Hence, the window of seed availability for postdispersal carabid weed seed predators could be considerably wide in time (Meiners, 2015). This would seem to suggest that finding weed seeds in time should not be an exacting task for carabid weed seed predators.

By contrast, the spatial overlap between weed seed shed (i.e., the seed shadow) and carabids is more complex. Some of the early carabid literature had proposed that carabid weed seed predators locate weed seeds upon random encounter (Cardina et al., 1996; Lovei & Sunderland, 1996). That is, if carabid beetles came across a weed seed, they would simply consume it. Beyond that, they would not spend considerable time and energy foraging for weed seeds that are less nutritious and more difficult to locate compared to prey (Kolb et al., 2007). We suggest that such assumptions in the literature have originated from the old “no‐choice” laboratory feeding bioassays, where carabids would accept the majority of food types offered to them (e.g., Shough, 1940). Such findings led carabid ecologists to assume that carabid species were scavengers or opportunistic feeders for the most part (Forsythe, 1982; Wheater, 1989). This idea still transcends the modern carabid literature, nonetheless. Recent evidence now points to random encounters as the exception rather than the rule, at least for carabid weed seed predators (Saska et al., 2019; Ward et al., 2014). The emerging evidence demonstrates that carabid beetles are likely to employ active foraging behaviors in search of weed seeds (Honek et al., 2007; Lundgren & Harwood, 2012). Carabid species in other words not only locate weed seeds in space but also show an ability to discriminate between seeds of different species and choose the most desired (Kulkarni et al., 2016). Therefore, finding weed seeds in space entails multiple exacting tasks for the foraging carabid beetle, especially with regard to seed detection and location.

### 3.2 Seed detection and location

Interactions between insects and plants that lead to weed seed predation should be viewed as a special case of plant–insect interactions (Jermy, 1984). The main difference here, though, is that the insects attack the reproductive units of the plant rather than its somatic tissues (McArt et al., 2013). It is well established that herbivorous insects rely on various types of visual and/or chemical cues to locate their host plants and assess their quality as sources of food (Bruce, 2015; Bruce et al., 2005). Chemical cues in terms of volatile and nonvolatile compounds have been proven to act as major drivers of host selection decisions in plant‐feeding insects (Eigenbrode & Esplie, 1995; Baldwin, 2010; Bruce & Pickett, 2011; Heil, 2014). By the same token, carabid weed seed predators are expected to rely on similar cue‐based systems for seed location and quality assessment (Kielty et al., 1996; Merivee et al., 2007, 2008).

Postdispersal weed seed predators have to search for seeds that are imbedded in complex, often cryptic environments (Aarstma et al., 2019; Forister et al., 2012). In other words, seed searching for carabid weed seed predators is a multilayered behavioral process fraught with challenges. Seed abundance is highly variable in both space and time (Dessaint et al., 1991; Henderson, 1990). Moreover, seeds are small in size and randomly scattered on the soil surface, or even buried underneath it (Menalled et al., 2007; White et al., 2007). This essentially renders any reliable cues for seed location and quality assessment sparse and difficult to detect (Baldwin, 2010; Poisot et al., 2011). Hence, successful foraging requires individual carabids to engage in active seed‐searching behaviors, vigorously scanning the environment for any seed‐related cues. Unlike random encounters, active foraging behaviors require the foraging animal to have highly developed motor abilities coupled with a sensitive sensory apparatus for picking up and recognizing cues of low detectability (Aarstma et al., 2019; Meiners, 2015). This assumption has been borne out as carabids have been shown to possess highly developed motor abilities along with a formidable arsenal of sensory receptors to guide their behaviors and food choice responses (Forsythe, 1983a; Merivee et al., 2000, 2008). Behavioral data on the whole indicate that food searching and food acceptance in carabid beetles, be that seed or prey, is an active and directed process guided by accurate sensory information collected from the environment (Harrison & Gallandt, 2012; Kulkarni et al., 2015b; White et al., 2007). We suggest that carabid predators should be viewed as active and selective foragers, clearly able to decipher between food types irrespective of random encounters.

### 3.3 Discrimination among different seed types and identifying the suitable seed type

The logic of optimal foraging theory assumes that food recognition and acceptance in selective foragers is guided by “search images” hardwired in the brain or learned from the environment (Garay et al., 2018; Krieger & Breer, 1999). Technically speaking, search images could be visual, chemical, or complex (visual and chemical) (Vet & Dicke, 1992). Assuch, carabid beetles as selective foragers are expected to employ search images to guide their food discrimination and acceptance decisions so that the most suitable food types are chosen for consumption. Such active decision‐making processes need accurate sensory sampling of the environment, and carabid species are thus expected to rely on their different sensory modalities (i.e., vision, olfaction, and gustation) to obtain the sensory information essential for food selection (Ploomi et al., 2003; Ramaswamy, 1988). Technically, gustation could be considered a special case of olfaction because olfactory and gustatory receptors show similar physiological structure and function, and collect chemical information of similar nature (Isono & Morita, 2010; Krieger & Breer, 1999). Henceforth, both smell and taste in carabid beetles will be treated as chemoperception in sections that follow.

It is still uncertain which sensory modality (vision or chemoperception) carabid species rely upon most to guide their food location and selection responses. Despite this uncertainty however, the evidence strongly hints that chemoperception is likely the top candidate for roles related to food searching and food choice in carabid predators (Law & Gallagher, 2015; Oster et al., 2014; Wheater, 1989). It can be proposed therefore that food selection decisions in carabid beetles, be that seed or prey, are generally guided by chemical cues encoding information that is interpreted based on innate or learned “templates” or “images” in the brain (Oster et al., 2014; Vet & Dicke, 1992). As such, chemical cues emitted from different types of weed seeds in arable fields are expected to mediate seed recognition and seed selection in carabid weed seed predators (Kulkarni et al., 2017b; Law & Gallagher, 2015; Lundgren et al., 2013). However, given the limited number of sensory studies and the highly diverse feeding habits of carabid species in arable fields, it cannot be ruled out that vision may also play some role in seed selection decisions, leading to the formation of complex search images in some cases (Dukas & Kamil, 2001; Wheater, 1989). This uncertainty stems from reports showing diurnal carabid species that actively hunt live mobile prey usually carry larger compound eyes and shorter antennae that house significantly fewer olfactory receptors compared to nocturnal species (Bauer & Kredler, 1993; Merivee et al., 2001, 2002). Such sensory differences seem to suggest that visual cues are likely to play more vital roles in guiding food searching behaviors in diurnal carabids compared to nocturnal ones (Wheater, 1989). The same studies showed that visually driven hunting behaviors in carabid predators broke down when prey items were immobilized. Therefore, it could be suggested that carabid visual receptors are more finely‐tuned toward detecting movement and are not expected to be of much help when carabid predators are searching for immobile and often cryptic food items like weed seeds (Srinivasan et al., 1999; Oster et al., 2014). Still, a possible role for vision in seed foraging in carabid weed seed predators cannot be decisively ruled out based on our current knowledge, and more studies are warranted.

Evidence in support of chemically mediated food detection, by contrast, is more abundant and comes from sensory and behavioral data pertaining to carnivorous and omnivorous carabids. Some carabid carnivores, for instance, showed positive orientation toward volatile chemicals originating from prey habitat (e.g., wheat extracts), as well as volatile chemicals specific to prey itself or to its pheromones (Kielty et al., 1996; Mundy et al., 2000; Tréfás et al., 2001). Moreover, electroantennographic detection (EAD) studies have shown that antennal preparation of *Pterostichus melanarius* Illiger adults could respond to odor chemicals originating from live or dead slugs, or even from the slug trails (McKemey et al., 2004). Likewise, antennal preparations of *P. melanarius* larvae produced detectable electrical signals when exposed to chemical odors of different prey types (Thomas et al., 2008). Strong electrical signals could also be recorded from labial palps of the ant‐specialist carabid *Siagona europaea* Dejean when exposed to formic and acetic acid secretions from its ant prey (Talarico et al., 2010).

Laboratory studies have also reported that some species of seed‐eating carabids exhibit olfactory‐oriented seed choice responses (Law & Gallagher, 2015). Positive orientation responses to seed volatiles were reported for some omnivorous carabid species when seed masses of different brassicaceous weed species were placed into odor chambers of a four‐arm olfactometer (Kulkarni et al., 2017b). The carabids used in the experiment also spent significantly more time in odor arms harboring the highly preferable weed species (i.e., *Brassica napus* L. seed in this case), indicating that they were able to discriminate among the seed species offered in the olfactometer based on odor alone. Moreover, the species of carabid weed seed predators were able to excavate weed seeds buried down to 1 cm below soil surface, without considerable loss in seed‐finding efficiency (Kulkarni et al., 2015b; White et al., 2007). Of note here, efficiency for excavating seeds that were buried at the same depth differed between the carabid species under study, yet seeds were detected and dug out in most cases. One can hence infer that differences in seed excavation efficiency resulted from species‐specific differences in soil‐burrowing responses between the tested carabids, not the absence of visual cues due to seed burial (Evans & Forsythe, 1985; Wallin & Ekbom, 1988). While there may be some role for vision in seed foraging, we suggest that any such role is likely minor given the findings of seed burial experiments. Overall, evidence to date clearly suggests that chemoperception is likely the primary mechanism guiding seed finding and seed selection responses in carabid weed seed predators.

It is crucial to reiterate that carabid predators forage in complex environments. Weed seeds are imbedded in an intricate matrix of environmental variables that could influence the foraging decisions of carabid predators in unpredictable ways (De Heij & Willenborg, 2020; Sarabi, 2019). Under such conditions, there will be certain cases where predicting the suitable seed type based on chemical cues alone fails to explain the observed seed selection responses of carabid weed seed predators (Foffova et al., 2020). That is, there will be certain cases where other impactful ecological factors might interfere and sway weed seed preferences in certain directions (De Heij & Willenborg, 2020). Factors relating to habitat properties, chemical or biophysical seed traits, taxonomic identity of the dominant species in the local carabid community, fear, and effects of learning and experience could all influence the processes of seed selection decision‐making, or perhaps override it altogether (De Heij & Willenborg, 2020; Ishii & Shimada, 2010). Overlooking such influential ecological and environmental factors is likely one main reason why field data involving seed predation remain ambiguous (e.g., Hatton et al., 2015; Honek et al., 2003; Petit et al., 2014). Therefore, we suggest that weed seed selection by carabid weed seed predators entails complex and multilayered processes that are unlikely to be explained by chemoperception‐based decisions alone. Hence, in sections that follow we will attempt to identify other possible ecological forces likely to shape seed selection decisions in carabid weed seed predators. The discussion will be reserved for analyzing functional traits for both weed seeds and carabid weed seed predators, and how their interactions might determine the predation strength under realistic situations. For a review of the possible ways in which the environmental factors influence weed seed predation dynamics, we direct readers to Kulkarni et al. (2015a), Sarabi (2019), or De Heij and Willenborg (2020).

## 4 BIOLOGICAL TRAITS POWERING THE ECOLOGICAL PROCESSES OF WEED SEED PREDATION BY CARABID BEETLES

### 4.1 Biological traits of weed seeds underlying vulnerability to predation risks

#### 4.1.1 Seed nutritional content

Seeds of plants usually contain large amounts of essential nutrients that are vital for embryo survival (Agrawal, 2007; Wang & Yang, 2020). Nutrients in seeds are typically comprised of carbohydrates (starch), protein, and fatty acids (lipids), and these show considerable variations across plant families and genera (Bretagnolle et al., 2015). It remains unclear which nutrient or combination of nutrients, if any, brings about a higher vulnerability to seed predation. Early studies with rodents reported that seeds containing high levels of protein suffered elevated predation rates (Gong et al., 2015; Halkier & Gershenzan, 2006; Henderson, 1990; Herms & Mattson, 1992). However, similar data for arthropod seed predators are scarce. Studies on ant‐dispersed seeds, for example, showed that seeds with lipid‐rich elaiosomes were usually picked up at higher rates by ants (Brew et al., 1986). Indirectly, this could be an indication of a lipid limitation in seed‐feeding arthropods because ants usually eat only the elaiosome and leave the seed intact (Brew et al., 1986; Rodgerson, 1998). In line with this, some carabid predators were found to interfere with ants and compete with them for seed elaiosomes (Ohara & Higashi, 1987). Carabid predators here consumed only the lipid‐rich elaiosomes and left the seeds intact in much the same way as ants. This could be an indirect indication that carabid predators also suffer lipid limitations in their diets and thus seek out lipids in seeds.

Recent field studies have indeed reported that weed seeds with high lipid content were usually more preferable to carabid weed seed predators than were seeds with low lipid contents (Petit et al., 2014). Laboratory feeding experiments also have shown that, within certain limits of seed size, weed seeds with high lipids suffered higher rates of attack by carabid weed seed predators (Gaba et al., 2019). Together, field and laboratory findings fall nicely in line with nutritional ecology data demonstrating that carabid species in arable fields usually forage under lipid‐limited conditions (Jensen et al., 2012; Raubenheimer et al., 2007; Toft et al., 2019). This agrees with other studies reporting that arthropod predators, including spiders and other insects, also suffer considerable lipid limitations in their natural habitats as well (Wilder & Eubanks, 2010; Wilder et al., 2013). Plus, carabids seem to maintain a strong preference for weed seeds even when offered along with prey items (Blubaugh et al., 2016; Frank et al., 2011; Lundgren & Harwood, 2012). Taken together, the totality of evidence thus far suggests that seeds of some weed species must contain certain lipids essential for carabid physiology, which might be absent from protein‐rich insect prey tissues (Booij et al., 1994; Wilder et al., 2013). Seeds could be thus essential to the diet of carabid weed seed predators. While it is compelling to assume that lipid limitations are one primary reason carabid weed seed predators consume seeds of certain weed species, much more detailed studies are needed to ascertain if other important nutrients like protein or carbohydrates might also impact seed selection decisions and thus, foraging ecology (Denno & Fagan, 2003).

#### 4.1.2 Seed chemical defenses

Plants cannot compromise on the nutritional needs for embryo survival, so they deploy different layers of defense that make seed nutrients difficult to access and costly to extract by seed predators (Rees & Long, 1992; Dalling et al., 2011). Plants, including weeds, mobilize a wide array of secondary metabolites to maturing seeds, many of which serve multiple defensive functions (Rattan, 2010; Trowbridge, 2014). While seed nutrients (i.e., primary metabolites) are likely to act as major feeding stimulants for carabid weed seed predators, the presence of defensive chemicals (i.e., secondary metabolites) could act against the phago‐stimulatory effects of nutrients (Chapman, 1999, 2003). Presumably, the acceptability of weed seeds would be determined by the overall balance between primary and secondary metabolites in seed tissues (Agrawal & Fishbein, 2006; Bernays et al., 2004; Seric Jelaska et al., 2014). That is, weed seeds with low levels of chemical defenses should be more preferable to carabid weed seed eaters, perhaps irrespective of nutritional content. However, the assumption that insects choose to feed on plant tissues based on chemistry alone does not hold true in all situations. A meta‐analysis has shown that levels of secondary metabolites in plant tissues do not always determine the acceptability of those tissue to insect herbivores (Carmona et al., 2011). This should not be surprising because the levels of primary and secondary metabolites in plant tissues are determined by complex interactions and tradeoffs between multiple plant traits (Blate et al., 1998; Davis et al., 2016). In line with this, studies that tested the impact of secondary metabolite levels on seed choice by rodent seed predators also produced conflicting results (Wang & Chen, 2012). Rodents, for example, avoided seeds with high levels of phenols in some cases (Gong et al., 2015; Henderson, 1990). In another more extensive study, effects arising from seed toxins on seed selection responses in rodent seed predators were totally absent (Rodgerson, 1998). More interestingly, studies investigating how the chemistry of weed seeds affect their persistence in seed banks showed that physical traits like seed mass, size, and coat hardness were much more important to long‐term seed persistence than chemical traits (Davis et al., 2008). Similarly, for seeds of multiple weed species, the physical characteristics of the seed were more crucial for avoiding predation by carabid predators than was seed chemistry (Foffova et al., 2020). We suggest that given the evidence to date, seed chemistry per se is unlikely to be the only driver of seed selection decisions in carabid weed seed predators. Instead, seed biophysical properties may profoundly interact with and perhaps even override the impacts of seed chemistry.

#### 4.1.3 Seed size and mass

Size is one physical trait of special interest to seed ecology (Baskin & Baskin, 1998; Dalling et al., 2020). Seed size directly relates to many seed quality parameters (Petit et al., 2014; Wang & Yang, 2020). Larger seeds, for instance, usually contain more energy and nutrients (Gong et al., 2015), but could also contain more chemical defenses (Agrawal & Fishbein, 2006; Wang & Chen, 2012). Also, larger seeds tend to have thicker and harder seed coats (Moles et al. 2003a; Moles et al. 2003b). Overall, the relationships between seed size, seed chemistry, and seed physical properties are quite complex and not well understood (Dalling et al., 2020; Wang & Chen, 2012). Nonetheless, the size of weed seeds was observed to be among the factors determining which seeds of weed species would be more preferable to carabid weed seed predators in laboratory trials (Lungren & Rosentrater, 2007; Saska, Honek, & Martinkova, 2019). In a laboratory study, Honek et al. (2007) produced measurements of dry mass for 25 different weed species and the body mass of 30 carabid weed seed predators in laboratory experiments. The authors managed to establish seed‐predator mass‐ratio curvilinear scaling relationships that strongly influenced seed selection responses of the carabid predators under study. Such mass‐scaling relationships have been shown to be vital for determining the strength of predator–prey interactions in vertebrate systems (Freeman & Leman, 2008), but the mechanistic aspects of size‐based prey choice remain poorly understood. Likewise, mechanisms of the size‐based seed selection responses in carabid weed seed predators remain unknown. More studies are needed in this regard to elucidate the factors underlying mass‐ratio scaling relationships and their impact on interaction strengths between weed seeds and carabid weed seed predators.

#### 4.1.4 Seed coat hardness

Seed coat hardness is determined by the amount of sclerenchyma deposited in the palisade cells of seeds (Radchuck & Borisjuk, 2014). Weed seed coat hardness, in general, decreases with increasing seed mass (Janzen, 1969; Lungren & Rosentrater, 2007). Yet, the opposite relationship patterns were observed for some species (van der Meij & Bout, 2000). Functionally, the relationship between seed mass and seed coat hardness differs from one weed species to another and is more likely to follow phylogenetic patterns rather than simple general linear patterns (Lovas‐Kiss et al., 2020). Despite these species‐specific differences, what seems to be of most consequence to the carabid weed seed predator is seed coat hardness (Jorgensen & Toft, 1997; Petit et al., 2014). The authors of two field studies observed that weed seeds with soft coats were much more susceptible to carabid weed seed predators than seeds with hard coats (Jorgensen & Toft, 1997; Noroozi et al., 2012). This piece of evidence remains anecdotal because neither study tested the effects seed coat hardness on removal rates of weed seeds. More direct testing of the relationship between seed coat hardness and predation avoidance has found that seed coat hardness was crucial in determining the susceptibility of weed seeds to predation by carabids (Foffova et al., 2020; Lungren & Rosentrater, 2007). This suggests that weed seed coat hardness could decisively determine vulnerability to seed predation if coat hardness differs considerably between the seed types in any given environment (Foffova et al., 2020). Still, it remains unclear if coat hardness acts alone or together with other seed traits as syndromes influencing predation avoidance (Agrawal & Fishbein, 2006; Dalling et al., 2011). A more plausible scenario is that the relative importance of any given seed trait with regard to predation avoidance is likely to be determined by how other seed traits also match or mismatch with the functional traits of the predator (Dalling et al., 2020; Foffova et al., 2020). In other words, trait values for the different seed defensive traits are only one part of the interaction. Predation pressure would also be determined by how many seed defensive traits actually match or mismatch with the functional traits of carabid predators with regard to the ability of the carabid to neutralize seed defenses (Ball et al., 2015; Quieter et al., 2007). This appears crucial because morphological traits of the seed like seed mass, size, and coat hardness undergo considerable physiological changes over time in the soil seed bank, and these changes are likely to affect seed vulnerability to carabid predation (Martinkova et al., 2016; Saska et al., 2019, 2020). For a summary of the different seed traits influential on weed seed vulnerability to carabid predators, see Table 2.

**TABLE 2:** Summary of potential weed seed traits that mediate seed vulnerability or avoidance to carabid weed predators

Seed trait	Vulnerability to seed predation	References
Seed nutrients (lipids)	+	Petit et al. (2014); Gaba et al. (2019)
Seed chemical defenses	0	Foffova et al. (2020)
Seed size and mass^a^	±	Lungren and Rosentrater (2007); Honek et al. (2007); Saska, Honek, and Martinkova (2019); Foffova et al. (2020); Saska et al. (2020)
Seed coat hardness	−	Jorgensen and Toft (1997); Lungren and Rosentrater (2007); Noroozi et al. (2012); Foffova et al. (2020); Saska et al. (2020)

Note

+, indicates a positive effect; −, indicates a negative effect; 0, indicates no documented effect.

^a^ Net effects depend on the seed‐predator mass‐ratio scaling relationship.

### 4.2 Biological traits of carabid predators of importance to weed seed feeding

#### 4.2.1 The physiological traits of carabid predators

Through feeding interactions, carabid predators obtain nutrients to address the nutritional limitations they face in their habitats and thus fulfill the nutritional needs for survival and reproduction (Frank et al., 2011; Potter et al., 2018). Generally speaking, protein and lipids are the two major macronutrients that drive the foraging efforts of arthropod predators (Jensen et al., 2012; Schmidt et al., 2012; Wilder et al., 2019). Weed seeds offer both types of macronutrients, but to different extents (Bretagnolle et al., 2015). Given that the majority of carabid species generally suffer lipid limitations in their agricultural habitats, foraging for lipids could be, in fact, the main trophic link binding weed seeds and carabid seed predators together (Gaba et al., 2019; Raubenheimer et al., 2007; Toft et al., 2019). Although the principal goal of trophic interactions is obtaining nutrients from the environment, there are other aspects of nutrient foraging behaviors than food extraction per se (McArthur & Pianka, 1966; Ydenberg et al., 1994). Setting out on foraging bouts is associated with different risks and entails high costs related to food handling and processing (Schoener, 1971; Pyke et al., 1977). Carabid weed seed predators in this sense need not only to find weed seeds, the chosen seed type or seed patch must also offer high rewards at a low cost (Fawki et al., 2003; Sih, 1984). Clearly, the balance between reward and cost of different seed types is likely a major factor shaping seed selection decisions (Bretagnolle et al., 2015; Brousseau et al., 2018). Moreover, how rewarding a certain type of weed seed is to any given carabid is determined by the biophysical and biochemical functional traits of the predator itself (Brousseau et al., 2018; Evans & Forsythe, 1985; Forsythe, 1987). In other words, the carabid predator needs to be able to neutralize seed defenses in order to gain more sufficient nutrition at a low cost. Should this not be the case, the carabid predator will suffer great fitness costs (Emlen, 1966; Pyke et al., 1977). Therefore, the species‐specific functional traits of carabid species are expected to strongly affect the types of weed seeds they come to accept for consumption. Among the various functional traits of carabid species, physiological and biochemical traits remain the least understood. Hence, it could be much more informative to examine the feeding ecology of carabid predators from a biophysical standpoint.

#### 4.2.2 The biophysical traits of carabid predators

The relationship between morphological traits and feeding habits is well established for insect herbivores, as well as for carabid predators (Bernays, 1991; Knapp & Knappova, 2013; Knapp & Uhnava, 2014). Curiously though, these relationships have remained largely overlooked in the study of carabid feeding ecology. For instance, there are generally strong functional links between mouthpart structure and the type of foods adult carabids can consume (Forsythe, 1982, 1983b). Recently, similar functional links between mouthpart morphology and the degree of feeding specialization have been uncovered for carabid larvae as well (Sasakawa, 2016). By and large, mouthpart morphology and structure of carabid species (both adults and larvae) seem to be among the fundamental functional traits that determine key aspects of the feeding specialization niche in terms of carnivory, omnivory, or granivory (Evans & Forsythe, 1985, Forsythe, 1987; Paarmann et al., 2006). Within each of these feeding niches however, functional traits other than mouthpart structure seem to determine aspects of food choice and preference.

Predator body mass allometry was found highly predictive of prey selection decision in carnivorous carabids preying on insects like aphids, collembola, and caterpillars in the field (Bell et al., 2008; Rusch et al., 2015; Schmitz, 2007). Similarly, predator body mass strongly affected seed selection decisions in carabid weed seed predators in laboratory studies (Honek et al., 2007; Martinkova et al., 2019; Saska, Honek, & Martinkova, 2019). Despite their strong impact, effects emerging from predator body mass on food choice remain poorly understood. Body mass is a complex trait with links to multiple other traits that directly relate to foraging behaviors, food handling capabilities, and functional responses of carabid predators (Aljetlawi et al., 2004; Brose, 2010; Reum et al., 2018). In this respect, a positive scaling relationship was found between carabid body mass (but not body length) and size of the muscle mass that power the mandibles and control the strength of bite force (Evans & Forsythe, 1985; Wheater & Evans, 1989).

The relationship between body mass, jaw musculature, and bite force has been found to shape vital aspects of feeding ecology in mammalian predators (Freeman & Leman, 2008; Wore et al., 2005), and granivorous birds (van der Meij & Bout, 2004). The importance of body mass and bite force for the feeding ecology of insects remains poorly understood, nonetheless. Emerging evidence in this regard suggests that body mass and bite force underlie fundamental aspects of the feeding ecology of predatory insects as well (Weihmnann et al., 2015; Blanke et al., 2018). Recent studies, for instance, have shown that match between bite force and cuticular hardness of prey was the most powerful factor in predicting prey preferences when carabid species were offered different types of prey (Brousseau et al., 2018; Konuma & Chiba, 2007). There are no similar studies looking into the role of bite force in seed selection decisions in carabid weed seed predators as yet. Besides, some authors speculate that bite force should play a key role in weed seed selection as well (Brousseau et al., 2018). Here, it is tempting to infer that the strong effects of carabid body mass in shaping the feeding response of carabid predators are most likely derived from its intimate relationship with bite force. This reasoning may explain why the relationship between carabid body mass and metabolic rates provided only a partial explanation of the interaction strength between carabid predators and prey (Brose et al., 2006, 2008; Brown et al., 2004; Reum et al., 2018).

The bio‐morphological reasoning laid out above seems to apply to larval carabids as well. Larvae of carabid weed seed predators, for example, have been shown to deliver stronger bite forces compared to strictly carnivorous larvae (Brandmayr et al., 1998; Paarmann et al., 2006). This is essential adaptation for handling the hard coats of weed seeds. Moreover, the feeding niche of carabids larvae expand as they grow and molt from one instar to another (Klimes & Saska, 2010; Saska, 2005). Such changes in feeding habits are likely due to increases in body mass and strength of mandibular muscles of the growing larvae as they advance from one instar to the other (Refeseth, 1984; Sasakawa, 2016). Overall, bio‐morphological traits are better in predicting the feeding ecology of carabid beetles compared to physiological traits (Bell et al., 2017).

Gape size should not be expected to constrain food choice in carabid species in general, including weed seed predators, because larval and adult carabids are fragmentary feeders and show no swallow feeding habits in general (Brousseau et al., 2018; Evans & Forsythe, 1985). Based on evidence discussed above, it is plausible to suggest that bio‐morphology in terms of mouthpart structure and bite force are two traits that shape key aspects of feeding specialization in carabid species, including carabid weed seed predators. Again, this accentuates further our pervious discussion as to why seed selection is unlikely to be based on seed chemistry alone. For a summary of carabid traits likely to affect ability of carabid predators to destroy weed seeds, see Table 3.

**TABLE 3:** Summary of potential carabid traits that influence the ability of carabid predators to destroy weed seeds

Carabid trait	Ability for weed seed destruction	References
Mouthpart morphology	+	Forsythe (1982, 1983b, 1987), (1985); Sasakawa (2016)
Body mass	+	Honek et al. (2007); Martinkova et al. (2019); Saska, Honek, and Martinkova (2019)
Bite force	+	Evans and Forsythe (1985); Wheater and Evans (1989); Brousseau et al. (2018)
Body length	0	Kulkarni et al. (2016)
Gape size	0	Evans & Forsythe (1985); Brousseau et al. (2018)

Note

+, indicates a positive effect; −, indicates a negative effect; 0: indicates no documented effect.

## 5 TRAIT‐BASED SEED CHOICE, FORAGING STRATEGIES, AND EFFECTS OF CARABID WEED SEED PREDATION ON WEED COMMUNITIES

The discussions above have established that seed detection and quality assessment are most likely mediated by olfactory mechanisms in some way. Following weed seed localization by a carabid seed predator, seed feeding will commence only if traits of both the predator and seed overlap to large extents (Ananthakrishan, 1990; Saska, Honek, Foffova, et al., 2019). Seed suitability rank in such trait‐based foraging scenarios will be determined by the ability of the predator to overcome the physical and/or chemical defenses of the seed (Foffova et al., 2020; Larios et al., 2017; Schmitz, 2007). The ability of the carabid predator to break through the seed coat is an essential first step before feeding on seed tissues can be initiated (Brousseau et al., 2018; Forsythe, 1982, 1983b). It can be expected therefore that in cases where predators are able to efficiently break through the seed coat of different seed types without considerable costs, seed chemistry will likely rule and predators would select seed types of superior chemical qualities (Blate et al., 1998; Moles et al. 2003a, 2003b). By contrast, if the different types of weed seeds show significant differences in seed coat hardness, predators would select the seed type that is easier to crush and handle, regardless of the chemical quality of that chosen seed type (Ananthakrishan, 1990; Potter et al., 2018). It is now more informative to build upon the trait‐based seed selection discussion and explore its aspects at the community level.

Weed communities in arable fields are composed of several coexisting weed species (Booth & Swanton, 2002; Petit et al., 2011). Consequently, weedy plants in any given weed community produce seeds of different types (i.e., species) and shed them in various numbers each year (Bagavathiannan & Norsworthy, 2012; Dessaint et al., 1991). There are thus two main sources of variability in weed seeds of arable fields; seed type as defined by species‐specific seed traits (i.e., trait‐based seed quality rank) and seed density, as defined by seed numbers per unit area (Albrecht & Auerswald, 2009). The optimal foraging theory predicts carabid predators, when choosing weed seeds, should choose be based not only on seed types but also seed numbers (Sih & Christensen, 2001).

The optimal seed foraging assumption predicts that carabid weed seed predators should keep track of any changes in abundance of seed types they prefer and adjust their foraging decisions accordingly (Hubbard & Cook, 1978; Pyke et al., 1977). Foraging decisions in carabid weed seed predators should therefore be dynamic and show changes through time and/or space. This assumption has been borne out by data from laboratory and field studies as carabid weed seed predators were shown to respond to changes in weed seed abundance in their environments (Dudenhoffer et al., 2016; Frank et al., 2011). In many cases, the rates of weed seed removal by carabid predators increased when seeds were offered in higher numbers per unit area (Honek et al., 2003; Westerman et al., 2008). Technically, these patterns suggest that carabid predators destroy more weed seeds as seed numbers increase and are therefore expected to have a stabilizing influence on weed populations as models of predator–prey dynamics suggests (e.g., Abrams, 2000; Sih, 1980, 1984). The mechanisms underlying the density‐dependent responses exhibited by weed seed predators in arable fields remain unknown, nonetheless. The phenomenon could arise from individual carabid predators consuming more weed seeds as they become more available per unit area, perhaps giving rise to foraging strategies ruled by functional responses (Holling, 1959). Alternatively, the higher rates of weed seed predation at increasing seed densities could come about by larger numbers of individual carabid weed seed predators consuming the dense seed patches, giving rise to foraging strategies ruled by numerical responses (Hulme, 1997; Marino et al., 2005). Some evidence has documented that dense seed patches attract higher numbers of carabid weed seed predators (Honek & Martinkova, 2001), but the correlation between carabid numbers aggregating to a dense seed patch and consumption rates of the preferable seed type in the patch was quite poor in some cases (Honek et al., 2005). Numerical responses are thus unlikely to always explain seed removal rates in responses to changes in seed density. Instead, both functional and numerical responses are more likely to work together and create complex seed predation dynamics in the field (Lester & Harmsen, 2002; Kuang & Chesson, 2010). Hence, a deeper treatment of seed foraging strategies requires elucidating how functional and numerical responses are shaped by changes in density of the preferable seed types relative to densities of other seed types (Lester & Harmsen, 2002).

It has been suggested that frequency‐dependent functions rule seed foraging strategies in arable fields (Greenwood, 1985; Horst & Venable, 2018). The key factor that powers seed predation dynamics in such case would be seed encounter rate (Kuang & Chesson, 2010). The seed type encountered most frequently in the environment will hence suffer the most attacks, whereas seed types that are less abundant will be largely ignored by seed predators (Merilaita, 2006). Frequency‐dependent models may help explain some aspects of weed seed selection in arable fields, but such explanations are based on seed numbers alone. Frequency dependence assumptions ignore that weed seeds shed in arable fields differ not only in number, but also in quality (Bagavathiannan & Norsworthy, 2012; Dessaint et al., 1991). Basically, frequency‐dependent reasoning turns weed seed predation interactions into a game of numbers, leaving no leeway for predators to choose seed types that suit them best as optimal foraging models predict (Pyke et al., 1977; Merilaita, 2006).

Ignoring this important shortcoming in frequency‐dependent models has led to wide contradictions in weed seed foraging data. For example, data from some field studies have shown that weed seed predation rates followed inverse‐density‐dependent patterns, and seed consumption rates declined as more seeds were offered per unit area (Cardina et al., 1996; Marino et al., 2005; Westerman et al., 2008). In such cases, weed seed predation would have a bio‐regulatory effect on weed populations only when weed seeds are scattered at low densities in the field. Higher weed seed densities would overwhelm the environment and saturate the functional and/or numerical responses of carabid weed seed predators, breaking down any bio‐regulatory effects against weed populations (Pannwitt et al., 2017; Petit et al., 2014). Intriguingly, the density‐dependent removal of weed seeds was totally absent in some feeding trials conducted under field conditions (Baraibar, Daedlow, et al., 2011; Noroozi et al., 2012; Pufal & Klein, 2013). The absence of density‐dependent effects in such situations suggests that factors other than seed numbers alone also play into seed selection decisions. Hence, weed seed predation interactions in arable fields are unlikely to be a mere numbers game.

Density‐dependent and selective (trait‐based) seed foraging should not be mutually exclusive in weed seed predation interactions as frequency‐dependent models presume (Baraibar, Carrion, et al. 2011; Mongel & Clark, 1986). Instead, the two strategies should work together, and abundance of preferable weed seed types would determine seed selection decisions in such cases (Pyke et al., 1977). One key difference from frequency dependence here is that upon an initial successful encounter with the preferable food type in the environment, the selective forager would alter its foraging behavior toward increasing the chances of coming across the preferable food type (Garay et al., 2018; Sih, 1980, 1984). Thus, food searching behavioral patterns exhibited by selective foragers should be directed rather than random, and not driven by encounter rates alone (Pyke et al., 1977; Hassell & Southwood, 1978). This fits nicely with the core assumptions of optimal foraging models as highly preferable weed seed types should always be selected when they are present in the environment (Sih & Christensen, 2001; Westoby, 1978). Less preferable types of weed seed would be consumed only when the highly preferable type is not available or is available only in small numbers (Hassell & Southwood, 1978).

**FIGURE 2:**
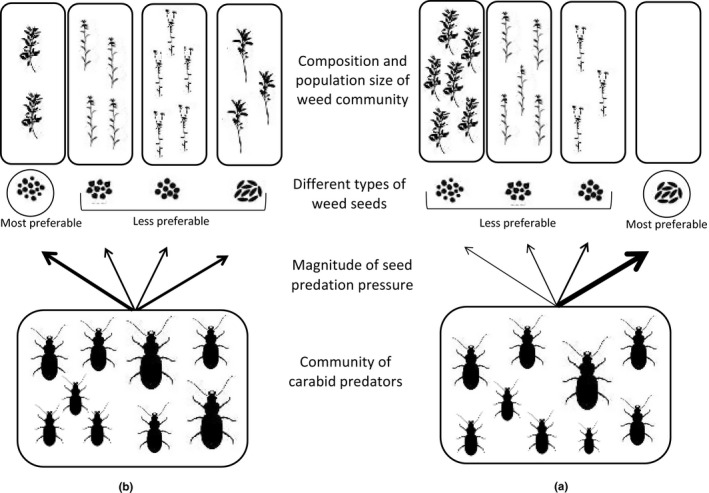
Possible scenarios of the foraging strategies of carabid weed seed predators and their effects on weed communities in arable fields. (a) the preferable type of weed seeds suffer higher attacks at lower densities, and switching to less preferable seed types takes place only carabid when the preferable seed type is depleted to large extents. Predation pressure is strongly directed against the preferable seed type leading to local extinctions in some case. (b) the preferable type of weed seeds suffer lower attacks at lower densities, and switching to less preferable seed types takes place when abundance of the preferable drops below a certain threshold. Predation pressure is more distrusted among the different seed types and no local extinctions take place

Given the above, it could be suggested that within certain ranges of seed densities, attack rates against the preferable weed seed type are likely to follow positive density‐dependent patterns (Cardina et al., 1996; Westerman et al., 2008). When the density of the highly preferable weed seed species starts to run low, the feeding responses of carabid weed seed predators can be summarized under the following two scenarios. The first scenario would involve seed predators continuing to attack the highly preferable weed seed type until it is depleted or no longer detectable (Holling, 1959; Zhang et al., 1992). Then, switching to less preferable seed types would take place. As a result, the highly preferable weed seed type would suffer significantly higher predation pressures when scattered in the environment at low densities compared to moderate and high densities. In such cases, the predation dynamics would be powered by inverse‐density‐dependent functions (Zhang et al., 1992). Under such conditions, the highly preferable weed seed type would suffer strong negative effects when its abundance is low, and its population could be extinguished in some local environments (Murdoch & Oaten, 1975; Figure 2a). There are field data to support the assertion of this scenario, suggesting that events of inverse‐density‐dependent weed seed predation could unfold under some realistic situations (see e.g., Cardina et al., 1996; Pannwitt et al., 2017; Westerman et al., 2008). Similar inverse‐density‐dependent prey selection patterns were also reported for carabid predators foraging on colonies of soybean aphids (Firlej et al., 2013). It could be inferred therefore that inverse density dependence might be the primary response governing foraging strategies in carabid predators, but more studies are needed before any conclusions can be firmly drawn here.

In the other possible scenario, carabid weed seed predators would switch their foraging mode toward the less preferable seed type when the abundance of the preferable type drops below a certain threshold level (Chernov, 1976; Pyke et al., 1977). Below that abundance threshold attacks against the preferable seed type starts to wane as predators start to seek more rewarding seed patches (Pyke, 1984; Sih, 1980, 1984). This would give the preferable seed type an “escape density” below which predation risks diminish (Sentis & Boukal, 2018). Local population extinctions would not be expected under such conditions. Instead, carabid weed seed predators could promote coexistence between multiple weed species (Juliano, 2001; Kuang & Chesson, 2010). This is a less attractive scenario for weed biocontrol programs because the predation pressure is more distributed among the different weed species in the community (Kuang & Chesson, 2010). But given that the abundance of the highly preferable weed seed species is kept below certain thresholds, some suppression against its populations should be expected (Holling, 1959; Lester & Harmsen, 2002; Figure 2b). Data in support of this scenario remain wanting; however, and studies testing this prediction are needed.

## 6 CONCLUSIONS AND FUTURE OUTLOOK

This review has provided evidence that the feeding ecology of weed seed carabid predators is quite complex. The diversity of ecological and environmental factors that influence strengths and dynamics of weed seed predation interactions is striking (De Heij & Willenborg, 2020; Kulkarni et al., 2015a). In this respect, mechanistic studies may provide good answers to why and how carabid weed seed predators target specific weed seeds. Knowledge of the core mechanistic aspects of weed seed predation ecology would be essential for future large‐scale studies. Mechanistic knowledge may help clarify where the line between ecological and environmental effects should be drawn. In this way, large‐scale studies could be better designed to elucidate the impact of agricultural practices on predation dynamics. Conservation biocontrol measures could then be tailored to enhance the functioning of carabid communities based on the local agricultural practices (Gurr & You, 2016; Headrick & Goeden, 2001). More detailed research studies would then identify the local carabid species that deliver substantial weed seed predation services in arable fields. Perhaps such keystone species of biological importance could then be listed to receive specific conservation measures so that their abundance and functioning in local environments is enhanced. In this way, local biodiversity could be enhanced while simultaneously providing critical agroecosystem services that improve agricultural sustainability.

Carabid communities in arable fields comprise numerous species, nonetheless. These communities show considerable changes in their species composition through space and time (Jacob et al., 2006; Pufal & Klein, 2013). Carabid species within those communities also are expected to interact, interfere, or even compete with one another in ways that remain poorly understood (Niemela, 1993; Niemela et al., 1997; Currie et al., 1996). Hence, the exact composition of the carabid community at any point in time or space would determine which exact weed species should incur the strongest predation pressures (Charalabidis et al., 2019; Petit et al., 2014). This paints a very complex picture for seed predation interactions at the community level, leaving the discussions laid out above rather simplistic. Notwithstanding, functional trait analysis studies offer powerful tools that may improve our ability to drill down through these complex layers and untangle the possible ecological forces driving weed seed predation dynamics at the community level (Rall et al., 2012; Reum et al., 2018). Along these lines, functional traits analyses of carabid communities found that average values of predator–prey mass‐ratio scaling parameters at the community level were key predictors of the suppression imposed by carabid predators against different prey types in the field (Roubah et al., 2014; Rusch et al., 2015). Similar trait‐based studies are likely to reach similar conclusions with regard to vital aspects of interactions between carabid weed seed predators and weed seed communities.

## CONFLICT OF INTEREST

Authors declare no conflict of interest.

## AUTHOR CONTRIBUTIONS

**Khaldoun A. Ali:** Conceptualization (equal); Methodology (equal); Resources (equal); Writing‐original draft (equal). **Christian J. Willenborg:** Funding acquisition (equal); Project administration (equal); Supervision (equal); Writing‐review & editing (equal).

## Data Availability

This work is a review of the findings published in the field of weed seed predation ecology and not based on collecting experimental data.
